# Differential gene expression in patients with subsyndromal symptomatic depression and major depressive disorder

**DOI:** 10.1371/journal.pone.0172692

**Published:** 2017-03-23

**Authors:** Chengqing Yang, Guoqin Hu, Zezhi Li, Qingzhong Wang, Xuemei Wang, Chengmei Yuan, Zuowei Wang, Wu Hong, Weihong Lu, Lan Cao, Jun Chen, Yong Wang, Shunying Yu, Yimin Zhou, Zhenghui Yi, Yiru Fang

**Affiliations:** 1 Division of Mood Disorders, Shanghai Mental Health Center, Shanghai Jiao Tong University School of Medicine, Shanghai, China; 2 Department of Neurology, Shanghai Renji Hospital, Shanghai Jiao Tong University School of Medicine, Shanghai, China; 3 Department of Genetics, Shanghai Mental Health Center, Shanghai Jiao Tong University School of Medicine, Shanghai, China; 4 Department of Psychiatry, Hongkou district mental health center, Shanghai, China; 5 Neurobiology Section, University of California, San Diego, CA, United States of America; University of Texas Health Science Center at San Antonio Cancer Therapy and Research Center at Houston, UNITED STATES

## Abstract

**Background:**

Subsyndromal symptomatic depression (SSD) is a subtype of subthreshold depressive and can lead to significant psychosocial functional impairment. Although the pathogenesis of major depressive disorder (MDD) and SSD still remains poorly understood, a set of studies have found that many same genetic factors play important roles in the etiology of these two disorders. Nowadays, the differential gene expression between MDD and SSD is still unknown. In our previous study, we compared the expression profile and made the classification with the leukocytes by using whole-genome cRNA microarrays among drug-free first-episode subjects with SSD, MDD and matched healthy controls (8 subjects in each group), and finally determined 48 gene expression signatures. Based on these findings, we further clarify whether these genes mRNA was different expressed in peripheral blood in patients with SSD, MDD and healthy controls (60 subjects respectively)

**Method:**

With the help of the quantitative real-time reverse transcription-polymerase chain reaction (RT-qPCR), we gained gene relative expression levels among the three groups.

**Results:**

We found that there are three of the forty eight co-regulated genes had differential expression in peripheral blood among the three groups, which are CD84, STRN, CTNS gene (F = 3.528, *p* = 0.034; F = 3.382, *p* = 0.039; F = 3.801, *p* = 0.026, respectively) while there were no significant differences for other genes.

**Conclusion:**

CD84, STRN, CTNS gene may have significant value for performing diagnostic functions and classifying SSD, MDD and healthy controls.

## Introduction

Depressive disorders affect about 10% of the population at some point in their life and is the leading cause of significant functional impairment and reduced quality of life, and known as a spectrum from subthreshold, minor to major episode. Unipolar major depressive disorder (MDD) is a pleomorphic mood disorder consisting of a cluster of depressive subtypes existing in a relatively homogeneous symptomatic clinical continuum, extending from subsyndromal symptomatic depression(SSD) through minor depressive episode, dysthymic disorder, major depressive episode and double depression. SSD is believed to be a clinically significant, inter-episode, depressive subtype of depressive disorders. Several studies have suggested that SSD was a transitory phenomenon in the depression spectrum. Although SSD have a smaller impact on quality of life than major depressive disorder[1], convergent evidence has identified that SSD is a common depressive status that affects different ethnic populations[2,3]. Several follow-up studies have suggested that SSD was a transitory phenomenon in the depression spectrum and was thus considered a subtype of depression[4].

Although the pathophysioloy of depression spectrum remain largely obscure, it has been reported that patients with SSD and MDD have similar family history and their first-degree relatives have a high risk of comorbility of depression and alcohol dependence, which implies that these two disorders could share identical genetic bases.

Up to now, more and more research has been conducted on the biological basis of MDD. MDD is found to be a complex disease, despite having a genetic basis, it does not conform to the classic Mendelian inheritance pattern. Linkage analysis was carried out in major depression to make it possible to identify candidate genes for this disorder. Interesting findings involves chromosome 11.2q33.34 chromosomal region and chromosomal region 17q11.2, Association studies showed that APOE, GNB3, MTHFR, SLC6A3 and SLC6A4 genes had a statistically significant association with major depression. Genome-wide association studies (GWAS) showed evidence of an association between an SNP polymorphism in the BICC1 gene (bicaudal C homologue 1 gene). Some study found that gene expression profiles could be used as a blood marker of MDD and careful independent validation has been carried out to prove their results[5].

At present, little research has been conducted on the biological basis of SSD and one of our studies clearly classifies SSD and MDD[6].Our previous study compared the expression profile and made the classification with the leukocytes by using whole-genome cRNA microarrays among drug-free first-episode subjects with SSD, MDD, and matched healthy controls (8 subjects in each group). Support vector machines (SVMs) were utilized for training and testing on candidate signature expression profiles from signature selection step. We tried different combination of signatures from the three pair-wise compartmental results and finally determined 48 gene expression signatures. The purpose of this study was to verify the differential expression of these genes in peripheral blood by means of quantitative real-time reverse transcription—polymerase chain reaction (RT-qPCR) in larger sample.

Further we correlate the selected gene with Hamilton severity depression and anxiety rating scales (HAMD and HAMA respectively) to know the somatic impact of depressive and anxious symptoms at both clinical and subclinical level in MDD and SSD patients.

## Material and methods

The study was conducted at the Division of Mood Disorders, Shanghai Mental Health Center, Shanghai Jiao Tong University School of Medicine between Jan 2009 and Dec 2011. Outpatients were recruited from the clinic and ward of Shanghai Mental Health Center. All procedures were reviewed and approved by Institutional Review Boards of Shanghai Mental Health Center. Written informed consent was obtained from each subject before any study-related procedures were performed.

### Subjects

Inclusion criteria for SSD group were: two or more depressive symptoms for at least 2 weeks with social dysfunction but without depressed mood or anhedonia, and having a total score of 17-itemHamilton Rating Scale for Depression (HAMD-17) from 8 to 16.Patients were included into MDD group who met DSM-IV criteria for MDD and had the total score of HAMD-17. Patients were excluded if they had substance dependence, severe medical illness, organic brain disease, pregnancy. Healthy control subjects have a score 7 or lower on the HAMD-17, and did not have any major Axis I disorders (including substance dependence, psychotic disorders, mood disorders and anxiety disorders), family history of mental disorder or severe physical diseases (hypertension, diabetes, cancer).

For the gene expression analysis, this study enrolled sixty drug-free Chinese Han patients with their first episode of subsyndromal symptomatic depression, sixty previously untreated patients presenting with their first episode of major depression disorder, and sixty healthy controls eventually. All subjects were screened by the Structured Clinical Interview for DSM-IV (SCID) and assessed through HAMD-17 and HAMA score by two experienced psychiatrists (inner coherence, Kappa = 0.87).

### Total RNA extraction, cDNA synthesis and reverse transcription

Total 40ml peripheral blood from MMD and SSD patients and healthy controls were collected during 7am to 9am without eating food. The blood was anticoagulant by the use of 2% ethylene diamine tetra-acetic acid (EDTA); then total RNA was extracted from 20ml blood with the QIA amp RNA blood Mini Kit (Qiagen, Chatsworth, CA, USA) and treated with DNase (Qiagen, Chatsworth CA, USA) from peripheral blood samples of patients and healthy controls. The complementary DNA (cDNA) was synthesized by incubating DNase-treated total RNA (1.0 μg) with Omniscript Reverse Transcription Reagents (Qiagen, Chatsworth, CA, USA) and a random primer according to the instruction manual. Purity of total cDNA in each sample was measured using 752 UV spectrophotometer, 1.8 < between 260nm wavelength and / 280nm wavelength UV absorbance the ultraviolet absorbance (of OD260 / OD280) <2. 0, RNA was concentration of 5 × 10–7 ~ 8 × 10–7 g / L, stored at—80°C refrigerator spare.

### Gene relative expression levels analysis

The target mRNA expression levels were measured by quantitative reverse transcription- polymerase chain reaction (RT-qPCR) using ABI Prism 7900 Sequence Tetection System (Applied Biosystems, CA, USA) with a 384-well format. For the RNA internal control, we used glyceraldehyde-3-phosphate dehydrogenase (GAPDH, Applied Biosystems, CA, USA) mRNA expression to normalize the target gene expression levels. The TaqMan Universal PCR Mastermix and TaqMan probes/primers were obtained from Applied Biosystems. Quantitative RT-PCR reaction was carried out as follow: 50°C (2 min) and 95°C (10 min), then 95°C for 50 cycles (10s), 59°C (1 min). Experiments were performed with triplicates for each sample.

Results of the real-time PCR data were represented as Ct value, defined as the threshold cycle of PCR at which a significant increase in the fluorescence signal is first detected. Data were collected and analyzed with Sequence Detector Software version 2.1 (Applied Biosystems). The Comparative Ct Values (ΔCt) was used for relative expression in target gene product, and 2–ΔCt represents the relative expression level. The ΔCt value of each sample (patients and controls) was obtained by subtracting the average GAPDH Ct value of each sample from the average target gene Ct value of each sample.

### Data analysis and statistical tests

Statistical analysis was performed by using the Statistical Package for Social Sciences (SPSS, version 17.0; Chicago, Ill). Demographic data were analyzed by using chi-square, ANOVA (one-way) respectively. ANOVA (one-way) followed by a post hoc LSD multiple comparison test was used to analyze the statistical difference of the co-regulated gene expression. P value less than 0.05 was considered statistically significant and all significant levels were two-tailed test. To correct for multiple testing, SAM analysis (Significance Analysis of Microarrays, Stanford University) was performed.

For the calculation of the Pearson’s correlation coefficient |r|, we used the Ct GENE/Ct GAPDH ratios and the scores of HAMD and HAMA as variables.

## Results

### Demographics

Demographic and clinical characteristics of the patients are shown in Table 1. There were no significant group differences between patients and controls for age and gender (*p>* .05).

**Table 1:** Demographic data for patients and healthy controls.

Group	Sex		Age(yr)	Course of disease(mo)
	Male	Female		
SSD	28	32	29.08±3.11	3.02±1.23
MDD	30	30	31.54±2.76	2.97±2.01
HC	30	30	30.46±3.62	none
*p*	>0.05	>0.05	>0.05	>0.05

Abbreviations: SSD = subsyndromal symptomatic depression; MDD = major depressive disorder; HC = healthy controls.

### Genes expression levels between the MDD, SSD and healthy control

By using quantitative RT-PCR, normalized by GAPDH, three target genes relative expression levels were significantly different among three groups, the results were listed as follows.

**Fig 1:**
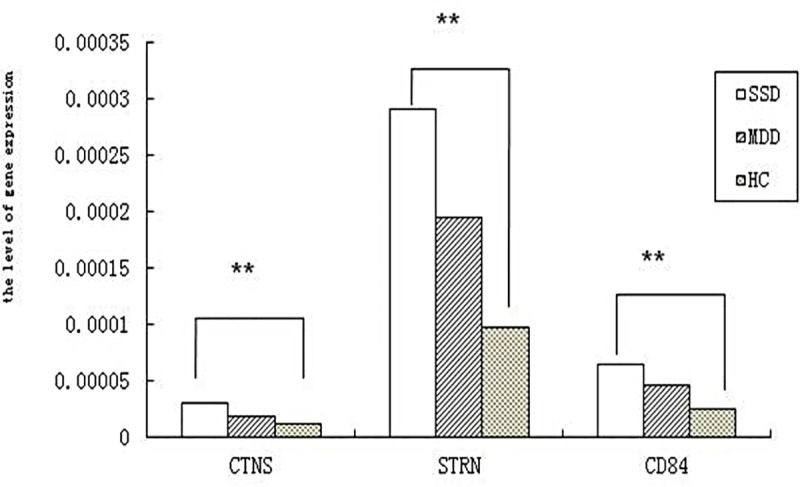
The gene expression was significantly different for CD84, CTNS and STRN gene in the three groups especially between SSD and health control group **P<0.05.

CD84 expression levels were significantly different relative expression levels among three group (4.59E-5±1.46E-5 in MDD group, 6.42E-5±8.64E-6 in SSD group, and2.41E-5±5.94E-6 in control group, F = 3.52, *p* = 0.03), CTNS expression levels were1.79E-5±4.78E-6 in MDD group, 2.91E-5±4.71E-6 in SSD group, and 1.06E-5±1.81E-6 in control group. STRN expression levels were 1.94E-4±6.19E-5 in MDD group, 2.90E-4±4.25E-5 in SSD group, and 9.64E-5±2.05E-5 in control group. There were significantly different relative expression levels for CD84, CTNSAND STRN among three groups (F = 3.53, *p* = 0.03; F = 3.38, *p* = 0.03; F = 3.80, *p* = 0.02, respectively), while there were no significant differences for other genes (PLoS ONE | www.plosone.org). ANOVA (one-way) followed by post hoc LSD multiple comparison test showed that compared with healthy controls, the relative expression levels of the three genes in both SSD and MDD were increased, furthermore, there were significant differences for CD84 and CTNS and STRN relative expression levels between SSD and control (SSD vs. controls *p* = 0.001; SSD vs. controls *p* = 0.002; SSD vs. controls *p* = 0.000 respectively). There were no differences for CD84 and CTNS and STRN relative expression levels between MDD and control (MDD vs. controls *p* = 0.453; MDD vs. controls *p* = 0.430; MDD vs. controls *p* = 0.383 respectively). The same results were found in CD84 and CTNS and STRN relative expression levels between MDD and SSD (MDD vs. SSD *p* = 0.64; MDD vs. SSD *p* = 0.28; MDD vs. SSD *p* = 0.50 respectively). (Fig 1)

Additionally, we correlate gene expression profiles with depression and anxiety severity scores and we found that gene CD84 was correlated with HAMD scale in item 10 named as Anxiety psychic (r = 0.318, *p* = 0.026) in MDD patients. In SSD patients gene CD84 was correlated with HAMD scale in item 7 named as Work and activities(r = -0.543, *p* = 0.024) (Data was shown in Tables 2 and 3 and S1 Table, S2 Table respectively).

**Table 2:** The relationship between depression/anxiety severity and genes expression profiles (STRN, CD84 and CTNS) in MDD patients.

Item of HAMD		STRNHs010 05318_m1 (N = 49)	CD84Hs0017 4668_m1 (N = 49)	CTNSHs001 91849_m1 (N = 48)
Depressed Mood	PC	-.061	-.029	-.068
	p	.815	.913	.802
Feeling of guilt	PC	.163	.081	.144
	p	.354	.435	.382
Suicide	PC	.023	.168	.110
	p	.931	.519	.685
Insomnia Early	PC	-.060	.027	.055
	p	.820	.917	.839
Insomnia mIddle	PC	-.106	.018	.044
	p	.685	.946	.871
Insomnia late	PC	-.027	.071	.067
	p	.919	.787	.806
Work and activities	PC	-.141	-.194	-.297
	p	.589	.456	.264
retardation	PC	.160	.322	.250
	p	.539	.207	.350
agitation	PC	-.301	-.340	-.384
	p	.241	.181	.142
Anxiety psychic	PC	-.450	-.543*	-.487
	p	.070	.024	.056
Anxiety somatic	PC	-.006	-.178	-.228
	p	.983	.494	.395
Somaticsymptoms:gastrointestinal	PC	-.307	-.250	-.320
	p	.230	.334	.227
Somaticsymptoms:general	PC	-.194	-.245	-.168
	p	.455	.343	.533
Genital symptoms	PC	-.333	-.311	-.277
	p	.192	.224	.298
hypochondriasis	PC	-.035	-.093	.002
	p	.894	.721	.993
Loss of weight	PC	-.446	-.398	-.394
	p	.073	.113	.131
Insight	PC	.063	.156	.017
	p	.809	.551	.950
HAMD T	PC	-.270	-.220	-.260
	p	.296	.397	.330

*: Correlation is significant at the 0.05 level (2-tailed).

Abbreviation: PC = Pearson Correlation; *p* = *p* value; HAMD T = HAMD total scores.

**Table 3:** The relationship between depression/anxiety severity and genes expression profiles (STRN, CD84 and CTNS) in SSD patients.

Item of HAMD		STRNHs010 05318_m1 (N = 49)	CD84Hs0017 4668_m1 (N = 49)	CTNSHs001 91849_m1 (N = 48)
Depressed Mood	PC	-.046	.061	.095
	p	.752	.676	.519
Feeling of guilt	PC	.008	.088	-.015
	p	.958	.549	.920
Suicide	PC	-.070	.059	-.114
	p	.635	.686	.439
Insomnia Early	PC	.043	.001	-.095
	p	.767	.995	.521
Insomnia mIddle	PC	-.161	-.122	-.212
	p	.270	.403	.148
Insomnia late	PC	-.043	.030	-.103
	p	.767	.838	.487
Work and activities	PC	.271	.318^*^	.158
	p	.060	.026	.207
retardation	PC	.059	.080	-.021
	p	.686	.583	.888
agitation	PC	.100	.136	.017
	p	.492	.350	.911
Anxiety psychic	PC	-.039	-.003	-.109
	p	.791	.984	.462
Anxiety somatic	PC	-.059	-.021	-.078
	p	.688	.888	.600
Somaticsymptoms:gastrointestinal	PC	.111	.066	-.024
	p	.449	.651	.869
Somaticsymptoms:general	PC	-.095	-.125	-.145
	p	.514	.392	.325
Genital symptoms	PC	-.081	-.049	-.073
	p	.579	.737	.620
hypochondriasis	PC	.109	.085	.039
	p	.457	.560	.791
Loss of weight	PC	.054	-.059	.003
	p	.713	.687	.985
Insight	PC	.268	.141	.167
	p	.063	.335	.257
HAMD T	PC	.093	.148	-.038
	p	.523	.310	.795

*: Correlation is significant at the 0.05 level (2-tailed).

Abbreviation: PC = Pearson Correlation; *p* = *p* value; HAMD T = HAMD total scores.

## Discussion

In this study, we mainly focused on the 48 differentially expressed genes and conducted Real time PCR analysis in the SSD, MDD and healthy controls. The results demonstrated the expression of CD84, STRN and CTNS were significantly altered among the three groups. Based on SAM analysis, the three genes remain significance after correction. Correlation analysis also found the anxiety severity item were positively correlated with CD84 gene expression. Interestingly, we found difference of three genes were specifically in the SSD group. Our results further support the evidence that the pattern of gene expression were no overlapping between MDD and SSD.

CD84 gene encodes a membrane glycoprotein that is a member of the signaling lymphocyte activation molecule (SLAM) family. This family forms a subset of the larger CD2 cell-surface receptor Ig super family. The encoded protein is a hemophilic adhesion molecule that is expressed in numerous immune cells types and is involved in regulating receptor-mediated signaling in those cells. Alternate splicing results in multiple transcript variants.

CTNS gene is lysosomal cystine transporter. Mutations in this gene cause cystinosis, a lysosomal storage disorder. Previously reported mutations include a 65-kb “European”deletion involving marker D17S829 and 11 small mutations. Doris A. Trauner, M.D. found that Children with cystinosis performed significantly more poorly on tests of visual Spatial and visual motor function than did controls. This deficit is associated with intact visual perception, but impaired spatial processing and memory[7,8], and is associated with deficits in mathematical skills[9]. Young children with cystinosis demonstrated a discrepancy such that non-verbal IQ indices (Performance IQ and Processing Speed Index) were significantly lower than verbal IQ indices[10]. The children with cystinosis performed significantly more poorly on visual spatial and visual motor measures than did controls, whereas visual perceptual skills remained intact.

STRN gene Locates in 2p22.2. It involved in plasma membrane estrogen receptor signaling, organism-specific biosystem. A small deletion in the 3’ untranslated region of striatin that leads to lower levels of striatin mRNA was recently implicated in a canine model of arrhythmogenic right ventricular cardiomyopathy[11]. What is more, the striatin gene was also found in one of twenty-two loci containing common variants associated with QRS interval length and cardiac ventricular conduction [12]. Striatin family members serve as molecular scaffolds that organize large signaling complexes.

The three genes involved in different signaling pathway. CD84 gene negatively regulates IgE high-affinity receptor signaling in Human Mast Cells[13]. It involes in immunoglobulin superfamily members. Some study show it is a homophilic receptor expressed on T cells, B cells, dendritic cells, monocytes, macrophages,eosinophils, mast cells, granulocytes, and platelets[14]. CD84 expression increases the following activation of T cells, B cellsand dendritic cells[15]. CD84 is also a hemophilic family member that enhances IFN-_ secretion in activated T cells. Some studies revealed that CD84 strongly self-associates with a Kd in the submicromolar range. CTNS gene mutation leads to an inherited multi-systemic disease resulting from failure of lysosomal cystine transport lysosomal cystine transport, STRN gene take part in Plasma membrane estrogen receptor signaling, organism-specific biosystem.The three genes belong to different pathway.

What was more, gene CD84 was negatively correlated with HAMD scale in item ten in MDD patients but not in SSD patients while gene CD84 in SSD patients was positively correlated with HAMD scale in item 7. It was an interesting phenomenon. The correlation of the expression of CD84 with scores of depression and anxiety in MDD and SSD patients raises the possibility that quantification of these gene transcripts in blood leucocytes could be used to explore the dimensional components of the stress load associated with depressive and anxious symptoms in different disease.

As for plasma levels, although we observed that the three gene plasma levels increased both in SSD and MDD patients compared with healthy controls, they could not distinguish between SSD and MDD patients.

### Applications

Three genes among 48 genes were identified in the independent sample population. Moreover, the three genes expression were only significantly associated with SSD. Thus, the three genes expression signatures may server as a potentially useful biomarker resource relating to SSD. In additions, this study also provided an contributory evidence on relationship between SSD and MDD.

### Limitations

There are several limitations in our study. Firstly, the sample size and the lower power were relative smaller. The larger sample SSD and MDD were utilized for replication the differentially expressed genes. Secondly, no function experiments with depressive animal model or in vitro cell lines were conducted with the three genes. Finally, the more evidences were required a better understanding of the relationship between SSD and MDD.

In our study, the differential expression of genes and their plasma levels only reflected the states in peripheral leukocytes, not in the central nervous system (CNS). As we all known, metabolism and regulatory processes of viable fresh brain tissues of subjects with psychiatric disorders are difficult to obtain, especially for brain tissue RNA studies, which not only complicates the analysis of a large quantity of patients, but also makes it difficult to have homogeneous samples with less confounding factors (such as age, gender, diet, clinic character, and psychotropic medications) [16]. Meanwhile, some studies recently showed that peripheral lymphocytes could reflect the metabolism of brain cells at certain extent[17,18]. Hence, peripheral blood lymphocytes have been considered an accessible and convenient neural probe of a number of cellular functions, including gene expression[19], and gene expression analysis of peripheral lymphocytes thus has been increasingly accepted as a potentially useful and convenient tool in detecting biomarkers of a variety of neuropsychiatric diseases[20,21,22]. Further study is needed to explore whether the change of gene expression levels in peripheral blood at baseline is trait or state marker. The findings should also be confirmed with replication studies in other populations.

## Supporting information

S1 Table The relationship between anxiety severity and genes expression profiles (STRN, CD84 and CTNS) in SSD patients* : Correlation is significant at the 0.05 level (2-tailed). Abbreviation: PC = Pearson Correlation; *p* = *p* value; HAMA T = HAMA total scores.
(DOCX)

S2 Table The relationship between anxiety severity and genes expression profiles (STRN, CD84 and CTNS) in MDD patients. *: Correlation is significant at the 0.05 level (2-tailed). Abbreviation: PC = Pearson Correlation; *p* = *p* value; HAMA T = HAMA total scores.
(DOCX)
