# Supplementary material for: Differential gene expression in patients with subsyndromal symptomatic depression and major depressive disorder
Source: PLoS One. 2017 Mar 23;12(3):e0172692. doi: 10.1371/journal.pone.0172692 (PMC5363801; doi:10.1371/journal.pone.0172692)
Supplement: S1 Table — : Correlation is significant at the 0.05 level (2-tailed). Abbreviation: PC = Pearson Correlation; p = p value; HAMA T = HAMA total scores. (DOCX) [file pone.0172692.s001.docx]

| **Table 4. The relationship between depression/anxiety severity and genes expression profiles (STRN, CD84 and CTNS) in SSD patients** | | | | |
| --- | --- | --- | --- | --- |
| **Item of HAMA** | | **STRNHs010**  **05318_m1**  **(N=49)** | **CD84Hs0017**  **4668_m1**  **(N=49)** | **CTNSHs001**  **91849_m1**  **(N=48)** |
| Anxious mood | PC | -0.047 | -0.132 | -0.127 |
|  | p | 0.723 | 0.876 | 0.781 |
| Tension | PC | 0.261 | 0.118 | 0.217 |
|  | p | 0.378 | 0.543 | 0.433 |
| Fear | PC | 0.029 | 0.267 | 0.219 |
|  | p | 0.871 | 0.653 | 0.763 |
| Insomnia | PC | -0.11 | 0.132 | 0.097 |
|  | p | 0.79 | 0.837 | 0.785 |
| Intellectual | PC | -0.124 | 0.132 | 0.076 |
|  | p | 0.679 | 0.857 | 0.786 |
| Depressed mood | PC | -0.033 | 0.127 | 0.079 |
|  | p | 0.886 | 0.679 | 0.714 |
| Somatic(muscular) | PC | -0.246 | -0.247 | -0.327 |
|  | p | 0.676 | 0.532 | 0.368 |
| Somatic(sensory) | PC | 0.265 | 0.431 | 0.327 |
|  | p | 0.547 | 0.324 | 0.436 |
| Cardiovascular symptom | PC | -0.424 | -0.453 | -0.439 |
|  | p | 0.325 | 0.271 | 0.241 |
| Respiratory symptom | PC | -0.467 | -.654 | -0.586 |
|  | p | 0.068 | 0.071 | 0.078 |
| Gastrointestinal symptom | PC | -0.017 | -0.237 | -0.348 |
|  | p | 0.967 | 0.476 | 0.379 |
| Genitourinary symptom | PC | -0.412 | -0.324 | -0.438 |
|  | p | 0.242 | 0.378 | 0.326 |
| Autonomic symptom | PC | -0.187 | -0.276 | -0.265 |
|  | p | 0.563 | 0.486 | 0.456 |
| Behavior at interview | PC | -0.421 | -0.439 | -0.329 |
|  | p | 0.232 | 0.326 | 0.326 |
| HAMA T | PC | -0.324 | -0.329 | -0.325 |
|  | p | 0.376 | 0.432 | 0.438 |
| *: Correlation is significant at the 0.05 level (2-tailed).  Abbreviation: PC= Pearson Correlation; *p*= *p* value; HAMA T=HAMA total scores. | | | | |
