# Supplementary material for: Differential gene expression in patients with subsyndromal symptomatic depression and major depressive disorder
Source: PLoS One. 2017 Mar 23;12(3):e0172692. doi: 10.1371/journal.pone.0172692 (PMC5363801; doi:10.1371/journal.pone.0172692)
Supplement: S2 Table — *: Correlation is significant at the 0.05 level (2-tailed). Abbreviation: PC = Pearson Correlation; p = p value; HAMA T = HAMA total scores. (DOCX) [file pone.0172692.s002.docx]

| **Table 5. The relationship between depression/anxiety severity and genes expression profiles (STRN, CD84 and CTNS) in MDD patients** | | | | |
| --- | --- | --- | --- | --- |
| **Item of HAMA** | | **STRNHs010**  **05318_m1**  **(N=49)** | **CD84Hs0017**  **4668_m1**  **(N=49)** | **CTNSHs001**  **91849_m1**  **(N=48)** |
| Anxious mood | PC | -.072 | -.147 | -.129 |
|  | p | .776 | .873 | .762 |
| Tension | PC | .257 | .125 | .233 |
|  | p | .436 | .338 | .471 |
| Fear | PC | .036 | .251 | .219 |
|  | p | .843 | .489 | .753 |
| Insomnia | PC | -.078 | .112 | .092 |
|  | p | .785 | .871 | .766 |
| Intellectual | PC | -.127 | .034 | .037 |
|  | p | .767 | .882 | .772 |
| Depressed mood | PC | -.156 | .112 | .101 |
|  | p | .862 | .698 | .759 |
| Somatic(muscular) | PC | -.237 | -.236 | -.338 |
|  | p | .674 | .557 | .339 |
| Somatic(sensory) | PC | .253 | .342 | .357 |
|  | p | .654 | .197 | .448 |
| Cardiovascular symptom | PC | -.439 | -.438 | -.477 |
|  | p | .337 | .211 | .239 |
| Respiratory symptom | PC | -.571 | -.476 | -.572 |
|  | p | .162 | .061 | .061 |
| Gastrointestinal symptom | PC | -.037 | -.238 | -.342 |
|  | p | .723 | .589 | .417 |
| Genitourinary symptom | PC | -.428 | -.344 | -.439 |
|  | p | .351 | .431 | .326 |
| Autonomic symptom | PC | -.237 | -.326 | -.275 |
|  | p | .547 | .446 | .489 |
| Behavior at interview | PC | -.443 | -.431 | -.364 |
|  | p | .237 | .342 | .327 |
| HAMA T | PC | -.325 | -.370 | -.317 |
|  | p | .285 | .452 | .469 |
| *: Correlation is significant at the 0.05 level (2-tailed).  Abbreviation: PC= Pearson Correlation; *p*= *p* value; HAMA T=HAMA total scores. | | | | |
